# Hypertensives' Knowledge About High-Sodium Foods and Their Behavior

**DOI:** 10.5935/abc.20160049

**Published:** 2016-05

**Authors:** Juliana de Fátima Teixeira, Maíra Ribas Goulart, Fernanda Michielin Busnello, Lucia Campos Pellanda

**Affiliations:** 1 Universidade Federal de Ciências da Saúde de Porto Alegre - UFCSPA, Porto Alegre, RS - Brazil; 2 Instituto de Cardiologia / Fundação Universitária de Cardiologia - IC/FUC, Porto Alegre, RS - Brazil

**Keywords:** Hypertension / mortality, Hypertension / prevention & control, Food Habits, Sodium Chloride, Dietary

## Abstract

**Background:**

In Brazil, the prevalence of systemic arterial hypertension (SAH) is approximately 30% of the total population. In 2010, SAH was the cause of death of about 9.4 million people worldwide. A healthy dietary pattern is important to maintain proper blood pressure levels and, consequently, disease control.

**Objectives:**

To describe the knowledge and practices of hypertensive patients cared for at a public hypertension outpatient clinic, and its relationship with high-sodium food.

**Methods:**

We applied a questionnaire to patients with questions related to sociodemographics, dietary pattern, frequency of ingestion of certain foods, and knowledge about their own disease.

**Results:**

We studied 221 patients, 56.1% of whom were women, and 53.8% had only elementary education. Their mean age was 57.7 ±13.5 years, and 75.6% of them reported having high blood pressure, and 11.3%, diabetes mellitus. Regarding dietary pattern, 62% used ready-to-use seasonings, but 94.1% reported not adding extra salt to their ready meals. Regarding patients' knowledge about high-sodium foods and SAH, only 8 patients had 100% of right answers, 37 patients had 73.8%, and 42 patients, 57% of right answers.

**Conclusion:**

Knowledge about SAH prevention and high-sodium foods was insufficient. Based on this study's findings, more effective educational strategies targeted at this population can be developed.

## Introduction

In Brazil, the prevalence of systemic arterial hypertension (SAH) is approximately 30% of the total population.^1^ Data have shown that, in 2010 only, SAH was the cause of death of around 9.4 million people worldwide.^2^ It is related to cardiovascular diseases, and almost 70% of individuals with acute myocardial infarction have increased blood pressure levels.^3^ The worldwide cost with cardiovascular diseases was estimated to be 906 billion dollars in 2015, and that figure is expected to reach 1,044 billion in 2030.^4^

The increase in SAH prevalence is due to both population increase and aging and risky behavior factors, such as smoking, alcohol intake, sedentary lifestyle, stress and unhealthy diet.^5^ It has been well established that excessive salt intake is associated with blood pressure elevation.^6^ In Brazil, the mean sodium intake is approximately 3.6 g for men (9 g of salt) and 2.7 g for women (6.7 g of salt), almost twice the recommended daily value of 1.5 g of sodium (3.7 g of salt).^7^

Lifestyle changes, such as healthy dietary habits, are important to maintain adequate blood pressure levels and to control the disease. Currently, communication media provide plenty of information on SAH; however, some studies have shown hypertensive patients to lack knowledge about their disease.^8-10^

Low levels of knowledge about SAH are associated with worse blood pressure control;^11^ thus, knowledge about their own disease, preventive care, experience exchange, and interaction between other effective methodologies allow hypertensive patients to have more effective self-care.^12-14^

Considering the need to establish educational programs according to characteristics specific to the social group approached, the present study aimed at describing the level of knowledge about SAH and its relationship with high-sodium foods, as well as the dietary habits of patients cared for at a multidisciplinary outpatient clinic mainly directed at patients with SAH.

## Methods

This cross-sectional study interviewed 221 patients who sought medical care at the Multidisciplinary Outpatient Clinic for SAH of the Instituto de Cardiologia (IC/FUC), Porto Alegre city, Rio Grande do Sul state. This study included patients aged at least 18 years, of both sexes, who provided written informed consent. Individuals with dementia or problems hindering adequate communication with researchers were excluded.

Sample size calculation considered a scenario with 50% of participants having adequate knowledge (worse scenario), 7% error margin and 95% confidence level. Thus, the need to include 200 patients was estimated. Considering a 10% margin of losses and refusals, we chose to include 220 patients.

To assess normality, the histograms of continuous variables, their measures of central trend and dispersion, and the Kolmogorov-Smirnof test were used. Non-paired Student *t* test and chi-square test were used to assess the differences between the total number of right answers and age group, disease duration, sex, schooling, marital status and ethnicity.

Data collection was performed before the visits to the multidisciplinary outpatient clinic. Patients completed a structured questionnaire with questions related to their dietary routine and dietary habits (intake of high-sodium foods, ready-to-use seasoning, additional salt to ready meals, light salt, ready-to-use dressings and instant soups; frequency of the intake of charcuterie, canned food and soft drinks). In addition, information on the following was collected: sociodemographics, such as age, sex, marital status; work condition; schooling; lifestyle; smoking habit; alcohol intake; physical exercise practice; and preexisting pathologies.

Individuals were considered to be sedentary when not meeting the current recommendations for physical activity practice (150 minutes per week for adults and 300 minutes per week for adolescents).^15^

Regarding knowledge about salt-rich foods, patients were asked about their perception of the salt content of certain foods, such as ready-to-use salad dressings, industrialized snacks, cheese, salami, ham, vegetable preserves, and instant soups. Knowledge about SAH included questions regarding the existence of a strategy to prevent high blood pressure and whether that disease could be cured. The multiple choice questions were elaborated based on the Brazilian Guidelines for Hypertension and on a nutritional knowledge questionnaire previously validated for the Brazilian population.^16^

The *Statistical Package for the Social Sciences* (SPSS) software, version 18.0, was used for statistical analysis. Categorical variables were described as proportions, and continuous variables, as means and standard deviations. To assess the relationship between intake, knowledge and other factors, Student *t* test and chi-square test were used, with a p value < 0.05.

The present study was assessed and approved by the Ethics Committee on Human Research of the IC/FUC (protocol 4412/09).

## Results

We assessed 221 patients aged between 18 and 84 years, with predominance of the female sex (56.1%) and of the age group of 60 years and over (48.9%). Mean age was 57.7 ± 13.5 years, and major characteristics are shown in Table 1.

**Table 1:** Socioeconomic, demographic and behavior characteristics of hypertensive patients (n = 221)

**Variables**	**N = 221**	**%**
**Sex**		
Female	124	56.1
Male	97	43.9
**Age (years)**		
18 - 29	10	4.5
30 - 39	9	4
40 - 49	38	17.2
50 - 59	56	25.3
> 60	108	48.9
**Schooling**		
Incomplete elementary school	133	60.2
Complete elementary school	40	18.1
Complete middle school	35	15.8
Incomplete middle school	0	0
High school	13	5.9
**Marital status**		
Married	136	61.5
Widowed	39	17.6
Single	22	10
Separated	24	10.9
Others	0	0
**Number of children**		
None	21	9.5
1 - 3	130	58.8
4 - 6	57	25.7
> 7	13	6
**Work condition**		
Retired	139	62.9
Salaried	25	11.4
Unemployed	11	5
Freelance	29	13.1
Housework	17	7.7
**Smoking**	23	10.4
**Smoking duration (years)**		
**10 - 15**	7	3.2
**20 - 30**	10	4.5
**30 - 40**	6	2.7
**Physical activity practice**	63	29.17

Regarding knowledge about their own disease, 75.6% of the population sample reported having SAH, and 79.2% had a family history of SAH.

Regarding dietary habits, 62% and 66.5% of the patients used ready-to-use seasoning and dressings, respectively, and 62.9% had no instant soups. Regarding light-salt intake, 95.9% of the patients had none, and 94.1% added no salt to ready-to-use meals. Table 2 shows the intake frequency of industrialized foods and soft drinks.

**Table 2:** Frequency of the intake of industrialized foods and soft drinks of hypertensive patients (n = 221)

**Variables**	**Intake frequency n (%)**				
	**1x/day**	**1x/week**	**2 - 3x/month**	**1x/month**	**none**
Canned food	0 (zero)	38 (17.2)	33 (14.9)	110 (49.8)	27 (12.2)
Soft drink	21 (9.5)	79 (35.7)	29 (13.1)	36 (16.3)	18 (8.1)
Salami Sausage	2(9.9)	44 (19.9)	57 (25.8)	61 (72.9)	57 (27.1)
Canned sardine	1 (0.5)	8 (3.6)	13 (5.9)	108 (48.9)	86 (38.9)

**Figure 1:**
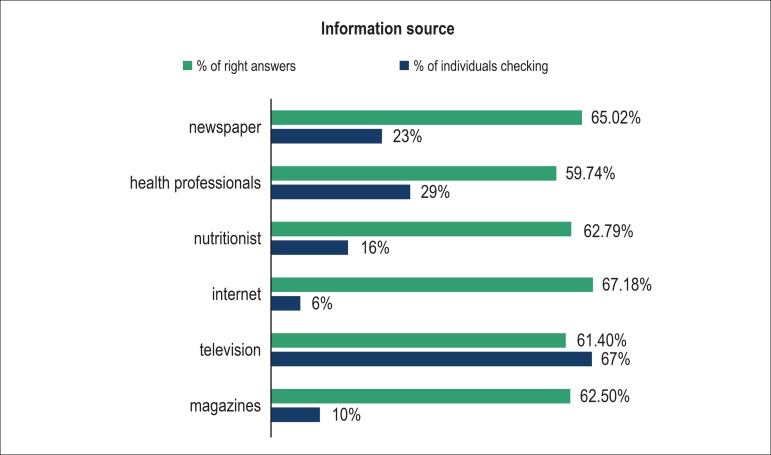
Information sources about foods and nutrition.

Figure 1 shows the information source about foods and nutrition reported by patients.

Table 3 shows the patients' knowledge about the high salt content in foods. Table 4 shows the number of right answers to the questionnaire according to the different characteristics of the sample. Significant differences were observed between the number of right answers and sex, information source and presence or absence of atherosclerosis. No difference was observed between the number of right answers and disease duration, ethnicity and age group (p > 0.05).

**Table 3:** Knowledge about the Intake of hlgh-sodlum foods of hypertensive patients (n = 221)

**Foods**	**Knew (n / %)**
Sausage	200 (90.5)
Industrialized snacks	200 (90.5)
Salami	195 (88.2)
Ready-to-use seasoning	172 (77.8)
Ham	130 (58.8)
Frankfurter	129 (58.4)
Vegetable preserves	127 (57.5)
Instant soups	116 (52.5)
Ready-to-use salad dressing	108 (48.9)
Mozzarella cheese	97 (43.9)
Canned foods	81 (36.7)

**Table 4:** Mean number of right answers in the questionnaire according to different characteristics

		**% right answers**	**p**
Sex	Female	62.81 ± 9.34	0.005
	Male	59.45 ± 10.09	
HDL-cholesterol	Normal	61.9 ± 9.83	0.01
	Altered	58.22 ± 9.34	
Atherosclerosis	With atherosclerosis	59.04 ± 9.24	0.004
	No atherosclerosis	62.63 ± 9.97	
Information source	Newspaper	65.02 ± 8.81	0.002
	No newspaper	60.27 ± 9.86	
Information source	Internet	67.18 ± 6.45	0.01
	No Internet	60.83 ± 9.9	
Dietary choice	Influence of flavor	59.45 ± 7.88	0.03
	No influence of flavor	62.01 ± 10.52	
Dietary choice	Influence of diet	65.17 ± 7.91	0.004
	No influence of diet	60.46 ± 9.99	
Dietary choice	Influence of healthy food	62.38 ± 9.53	0.03
	No influence of healthy food	59.88 ± 10	

Student t test

Table 5 shows the topic approached and the proportion of right answers for each question.

**Table 5:** Mean values of body mass Index (BMI) according to cholesterol levels, Information source and Influence on dietary choice

		**BMI (kg/m^2^)**	**p**
Cholesterol	Altered	32.34 ± 2.77	0.04
	Normal	33.14 ± 3.72	
Information source	Newspaper	31.81 ± 3.07	0.01
	No newspaper	33.09 ± 3.45	
Dietary choice	Influence of convenience	31.45 ± 3.63	0.000
	No influence of convenience	33.12 ± 1.32	

Student t test

When asked about the existence of any strategy to prevent SAH, 90.5% of the patients answered 'yes'. Regarding disease prognosis, 29.9% of the patients answered high blood pressure can be cured. However, 70.1% of the patients answered there is only disease stabilization or prevention.

There were 14 questions concerning patients' knowledge about high-salt foods and their relation to SAH (14 right answers = 100%). Only 3.6% of the patients scored 100%, the mean number of right answers being 8.9. No significant difference was observed between number of right answers and age group, disease duration, sex, schooling, marital status and ethnicity.

## Discussion

The present study shows an insufficient level of knowledge of hypertensive patients about SAH. Considering that those patients were followed up at a SAH outpatient clinic, the fact that one fourth of them did not acknowledge their own diagnosis of SAH is worth noting. These results are in accordance with the literature.^17^

Most participants used ready-to-use seasonings and dressings in their meals, increasing the daily amount of sodium in their diets. Sodium intake above the recommended values is directly related to blood pressure levels elevation.^18^ However, it is worth noting that more than 90% of the interviewed individuals did not add additional salt to their meals, confirming other studies.^5,17^ Nevertheless, a Brazilian study^12^ has shown that the mean sodium intake for men and women remains high.

Clearly, patients have difficulty properly addressing SAH prevention and/or control. That shows the need to instrument health professionals to both elaborate specific patient-directed strategies, and to promote dietary habits, aiming at providing hypertensive patients with autonomy when choosing their foods, with consequent improvement in their quality of life.

Primary prevention of SAH can be obtained through non-pharmacological treatment, mainly lifestyle changes, which include weight control, sodium and alcohol intake reduction, smoking cessation and regular physical activity practice.^19^

Knowledge about SAH is important to promote more effective self-care and to prevent worsening of the patients' clinical findings. Group activities have proved to be effective to build knowledge, because they comprise: interaction with health professionals, whose role is to facilitate teaching and to provide guidance according to the patients' understanding level; exchange of experiences between hypertensive participants.^11^ Thus, integrated health care contributes to reduce the SAH risk factors.

In accordance with other studies, in our sample women showed to have more knowledge about nutrition.^9^ This can be due to cultural issues regarding more interest in nutrition, or to the fact that women more often search for health care services and have more opportunities to discuss their issues.^9^ This could explain the higher prevalence of female patients in this sample.

That higher prevalence of the female sex, corroborating other studies with the same population profile,^16,20^^21^ leads us to assume women are more concerned with their disease and more often search for health care.

Regarding schooling, 60% of the sample had incomplete elementary education. This might have influenced adhesion to treatment or have hindered the understanding about SAH and the guidance provided by the health professionals about the disease. This might justify 25% of the participants reporting not having a diagnosis of SAH, despite being followed up at a SAH outpatient clinic. This points to the need for specific intervention strategies, directed to patients' profiles, to improve their knowledge about their disease.

The low prevalence of the smoking habit found in this study can be considered beneficial, because smoking can be associated with interruption of the SAH treatment.^22^ It is worth noting, however, that the instrument of data collection in this study did not comprise the "ex-smoker" category; therefore, ex-smokers were included in the "non-smoker" category. That fact is negative, because it involves reverse causality, that is, patients quitted smoking in an attempt to improve their health condition. Likewise, most patients denied the habit of alcohol intake, which has been found in other studies.^23^

This study had limitations, such as memory bias, which might have affected the accuracy of the responses, and the limitation inherent in cross-sectional studies, that is, the absence of follow-up over time, not allowing us to establish causal relationships. Therefore, a causal relationship with insufficient knowledge could not be directly established. However, knowledge is the first step to behavior change, and this study aimed at assessing that knowledge, contributing to support the elaboration of more effective education programs.

## Conclusion

Considering the insufficient knowledge about their own disease and the unhealthy dietary habits observed among hypertensive patients in this study, in addition to the sedentary lifestyle reported by most of them, strategies of nutrition education and health promotion should be developed, aimed at increasing their knowledge about their own disease and empowering them to self-care and lifestyle changes.
